# Delayed-Onset Infectious Aortic Aneurysm Caused by Hypermucoviscous Klebsiella pneumoniae Detected on Reevaluation Contrast Computed Tomography

**DOI:** 10.7759/cureus.91167

**Published:** 2025-08-28

**Authors:** Yukihito Nakamura, Manabu Hayakawa, Yoshihiro Ishii, Masato Edamoto, Daisuke Sakaguchi

**Affiliations:** 1 General Practice, Miyazaki Prefectural Miyazaki Hospital, Miyazaki, JPN

**Keywords:** contrast-enhanced computed tomography, hypermucoviscous klebsiella pneumoniae, hypervirulent klebsiella pneumoniae, infectious aortic aneurysm, klebsiella pneumoniae, string test

## Abstract

*Klebsiella pneumoniae *subsp. Pneumoniae (Kp) is a Gram-negative bacillus commonly encountered in clinical practice, often causing urinary tract infections and pneumonia. Kp includes classical strains (cKp) and hypervirulent strains (hvKp), the latter causing invasive syndromes, such as liver abscesses, endophthalmitis, and meningitis. HvKp is increasingly reported in East Asia, including Taiwan, and has occasionally been implicated in infectious aortic aneurysms (IAA). The hypermucoviscous phenotype of Kp (hmKp), identified by a positive string test, is strongly associated with hvKp. We report a case of sepsis caused by hmKp in a 65-year-old male. Although the patient initially responded to antibiotic therapy, low-grade fever and elevated inflammatory markers persisted. Reevaluation using contrast-enhanced computed tomography (CECT) revealed a delayed-onset IAA. The patient underwent emergency endovascular aneurysm repair (EVAR), followed by open aortic replacement (OAR), resulting in survival. This case highlights the importance of considering deep infections such as infectious aneurysms in patients with hmKp sepsis that fails to show expected clinical improvement despite appropriate treatment. Timely reevaluation with CECT is crucial for detecting life-threatening complications and enabling prompt, definitive management.

## Introduction

*Klebsiella pneumoniae *subsp. Pneumoniae (Kp) is a Gram-negative bacillus frequently encountered in clinical practice, typically causing urinary tract infections and pneumonia [1]. This species includes classical strains (cKp) and hypervirulent strains (hvKp), the latter responsible for invasive syndromes such as liver abscess, endophthalmitis, and meningitis [1]. HvKp strains are characterized by increased capsular polysaccharide production (notably K1 and K2 serotypes), enhanced siderophore activity, and virulence genes such as magA and rmpA [2]. The hypermucoviscous phenotype (hmKp), identified by a positive string test, is strongly associated with hvKp and often used as a surrogate marker of hypervirulence in clinical settings [3]. Reports of hvKp/hmKp infections have been increasing in East Asia, including Taiwan and Japan [4].

Infectious aortic aneurysm (IAA) is a rare but life-threatening condition, most frequently caused by *Staphylococcus aureus *or *Salmonella *species [5,6]. IAAs are characterized by rapid expansion and a high risk of rupture, and mortality remains high unless timely diagnosis and combined surgical and antimicrobial therapy are provided [7]. Although *K. pneumoniae *is an uncommon cause, hvKp and hmKp have occasionally been implicated in IAAs [8], underscoring the importance of recognizing this association in clinical practice.

Here, we report a rare case of delayed-onset IAA caused by hmKp, which was detected on re-evaluation imaging after persistent inflammation despite appropriate antibiotic therapy.

## Case presentation

A 65-year-old Japanese man presented to the emergency department with high-grade fever (41.8°C) and altered mental status. His past medical history included atopic dermatitis and ossification of the posterior longitudinal ligament. He reported daily alcohol consumption and a 47-year history of smoking 20 cigarettes per day since the age of 18.

On examination, his vital signs were as follows: blood pressure of 90/58 mmHg, heart rate of 94 beats per minute, respiratory rate of 30 breaths per minute, and SpO₂ of 100% on 6 L/min oxygen via face mask. He was alert with a Glasgow Coma Scale (GCS) score of 15. Physical examination was otherwise unremarkable.

Initial laboratory tests revealed marked leukocytosis and elevated C-reactive protein (CRP) levels. Non-contrast CT showed rectal wall thickening and extensive aortic calcifications. Lumbar puncture and head CT were unremarkable. Urinary cultures, sputum cultures, cerebrospinal fluid (CSF) cultures, and two sets of peripheral blood cultures were obtained, and empiric intravenous ceftriaxone (CTRX) was initiated for presumed sepsis of urinary or gastrointestinal origin.

**Figure 1:**
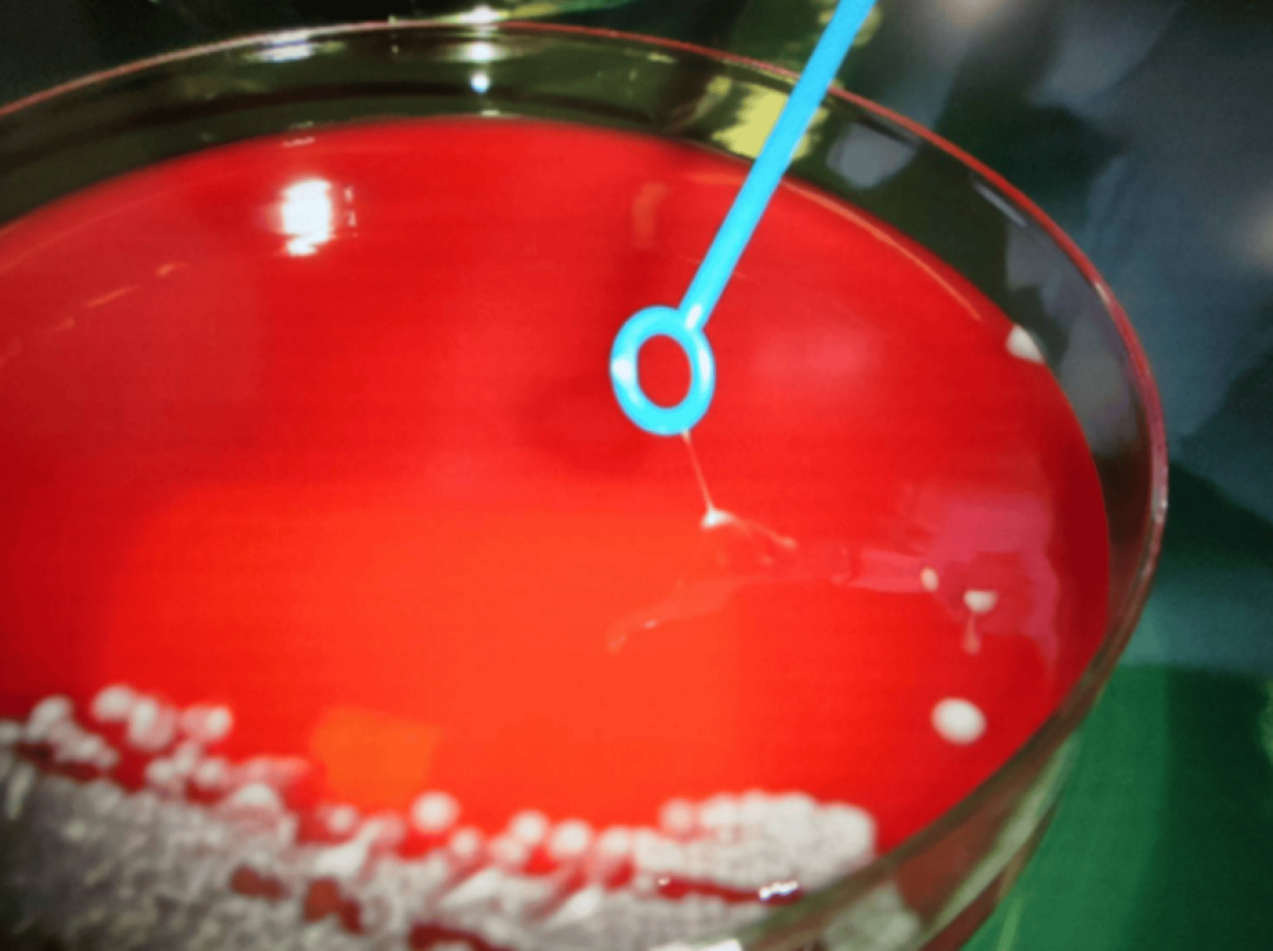
Positive string test for Kp A mucoviscous string measuring more than 5 mm was observed when a colony on blood agar was stretched with an inoculation loop, indicating a positive string test. This finding is characteristic of the hypermucoviscous phenotype. Kp: *Klebsiella pneumoniae*

On hospital day two, blood cultures yielded Kp. Considering the possibility of an extended-spectrum β-lactamase (ESBL)-producing strain, antibiotic therapy was escalated to meropenem (MEPM). The isolate tested positive on the string test, consistent with hmKp (Figure 1). Contrast-enhanced computed tomography (CECT) and ophthalmologic examination ruled out liver abscess and intraocular involvement. On hospital day four, antimicrobial susceptibility testing (AST) according to CLSI guidelines demonstrated that the isolate was not ESBL-producing. Although this result allowed de-escalation, MEPM was continued in view of the potential for occult deep-seated infection associated with hmKp and the favorable tissue penetration of MEPM.

**Figure 2:**
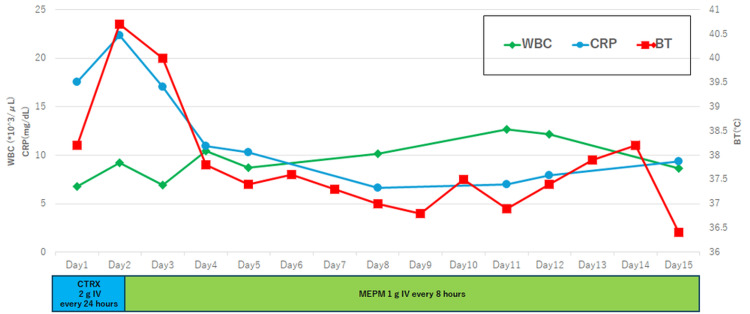
Clinical course of WBC, CRP, and BT during initial antimicrobial therapy WBC, CRP, and BT were monitored daily following the administration of intravenous CTRX and MEPM. Following escalation to MEPM on day two, a marked improvement in inflammatory markers was observed by day four. Dual Y-axes represent WBC/CRP (left) and BT (right). WBC: white blood cell count; CRP: C-reactive protein level; BT: body temperature; CTRX: ceftriaxone; MEPM: meropenem

**Figure 3:**
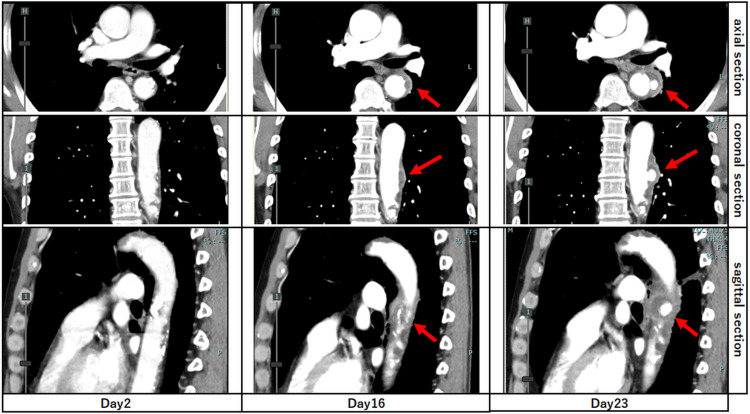
Serial CECT images demonstrating the rapid progression of an IAA Axial, coronal, and sagittal sections of CECT images on day two, day 16, and day 23. A saccular aneurysm of the descending thoracic aorta enlarged from 6 mm on day 16 to 13 mm on day 23 (red arrows). CECT: contrast-enhanced computed tomography; IAA: infectious aortic aneurysm

Although the patient’s vital signs stabilized, low-grade fever and elevated inflammatory markers persisted (Figure 2). A CECT on day 16 was performed to reassess the infectious focus and revealed a 6 mm expansion of the descending thoracic aorta. By day 23, the aneurysm had enlarged to 13 mm with an ulcer-like projection, strongly suggesting an IAA (Figure 3).

**Figure 4:**
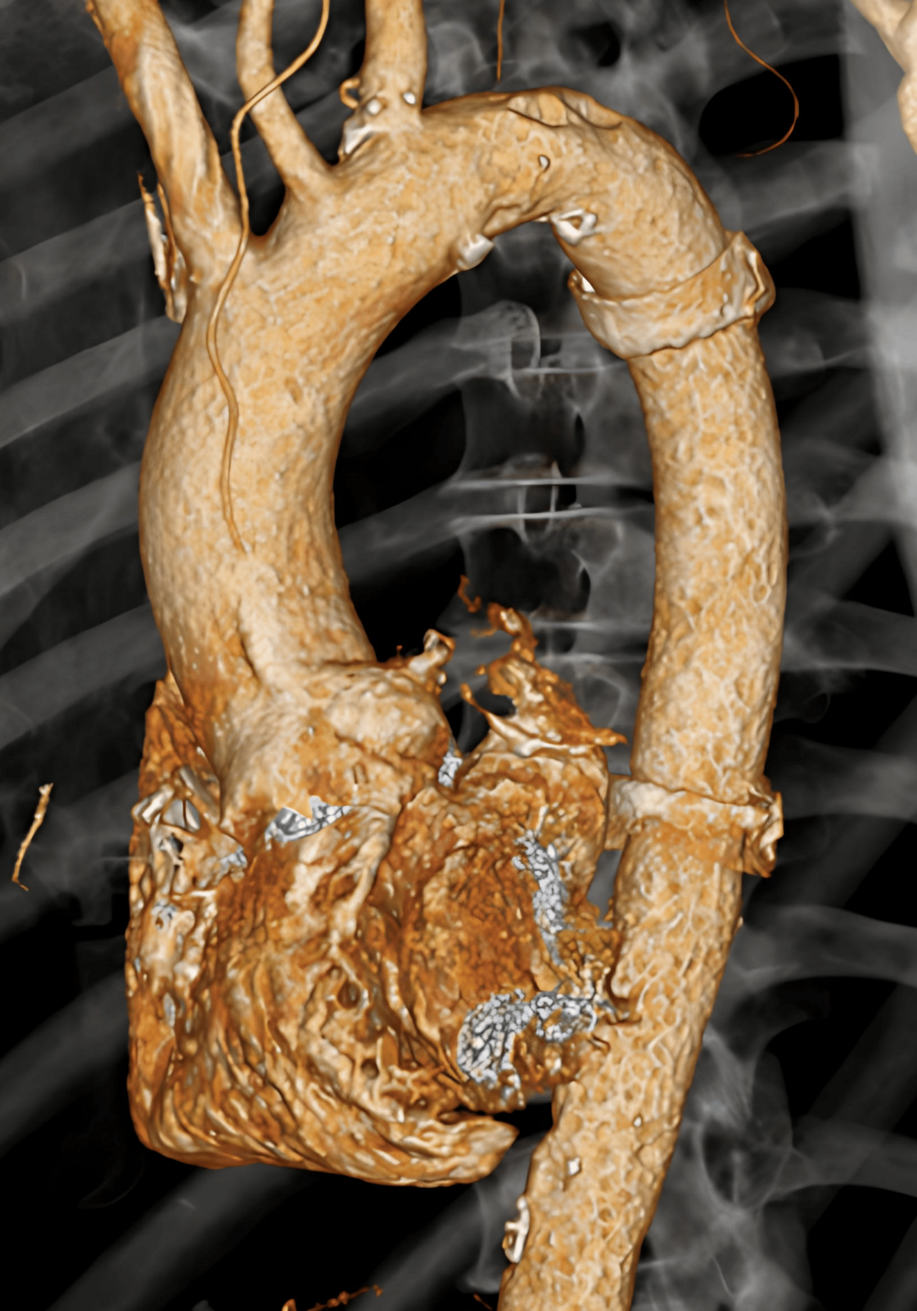
Postoperative 3D-CT of the thoracic aorta graft 3D-CT reconstruction obtained 31 days after symptom onset, following open surgical graft replacement, showing the thoracic aortic graft with no residual aneurysm. 3D-CT: three-dimensional computed tomography

**Figure 5:**
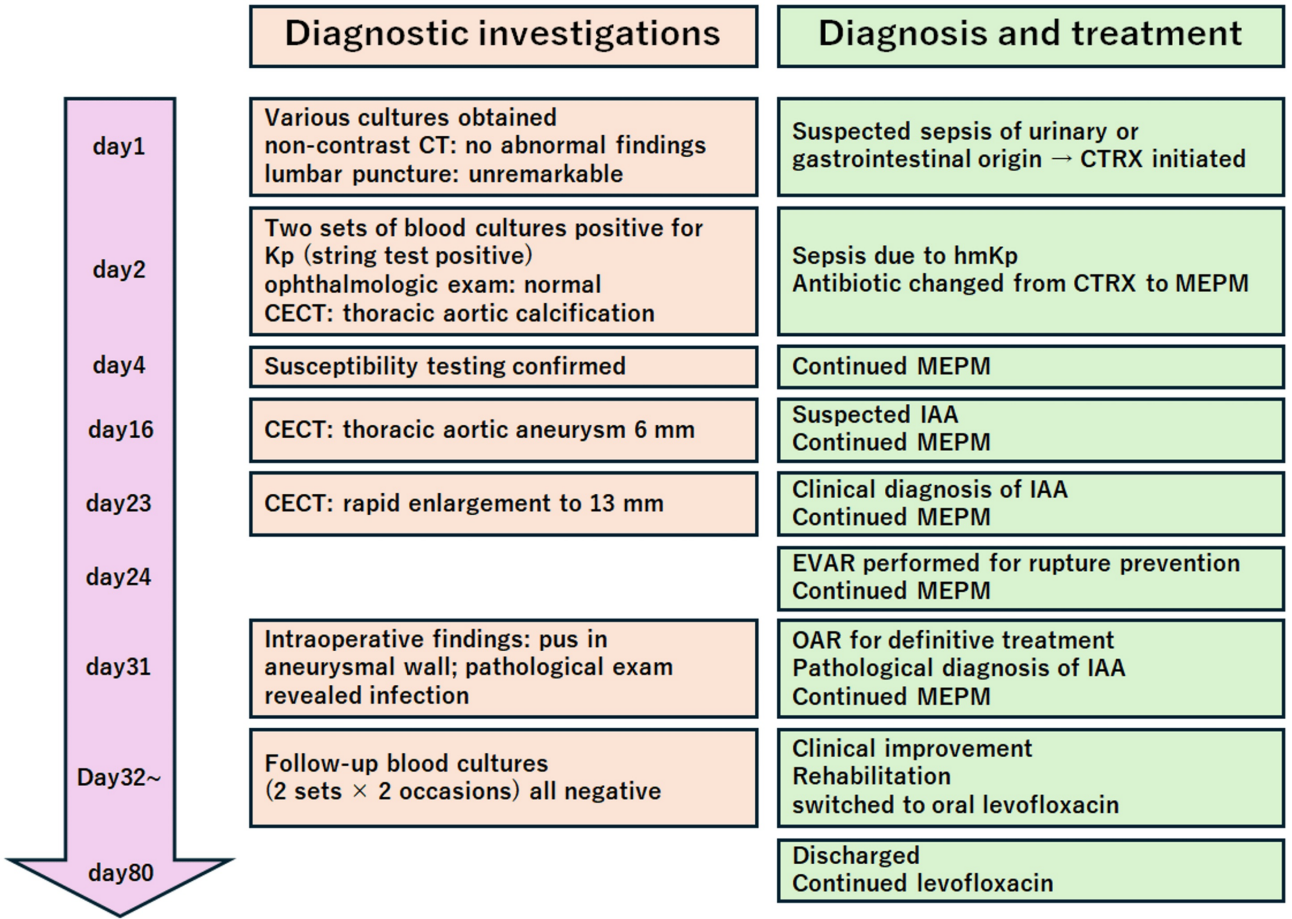
Timeline of diagnostic investigations and treatment The patient was admitted with suspected sepsis and initially treated with CTRX, which was later changed to MEPM after blood cultures grew hmKp. CECT initially showed only aortic calcification, but follow-up imaging demonstrated a rapidly enlarging thoracic aortic aneurysm, leading to the diagnosis of an IAA. EVAR was performed on day 24, followed by OAR on day 31, with pathological confirmation of infection. The patient gradually improved, was switched to oral levofloxacin, and was discharged in stable condition on day 80. CTRX: ceftriaxone; MEPM: meropenem; hmKp: hypermucoviscous *Klebsiella pneumoniae*;* *CECT: contrast-enhanced computed tomography; IAA: infected aortic aneurysm; EVAR: endovascular aneurysm repair; OAR: open aortic replacement

To prevent rupture of the rapidly enlarging aneurysm, endovascular aneurysm repair (EVAR) was performed on hospital day 24. However, EVAR has been reported to carry a higher risk of postoperative infectious recurrence compared with open aortic replacement (OAR) [9], and graft explantation is generally recommended if endograft infection occurs [10]. Therefore, for definitive treatment of the IAA, OAR was performed on hospital day 31 (Figure 4). Macroscopic pus was observed in the aneurysm, although tissue cultures and Gram staining were negative. Histopathological examination of the aortic wall revealed decreased or lost elastic fibers in the outer one-third of the media, replaced by inflamed granulation tissue or loose fibrous tissue with lymphoplasmacytic and neutrophilic infiltration. Considering these findings in the context of the clinical course, the IAA was diagnosed as being caused by hmKp. The postoperative course was uneventful, and the patient recovered without recurrence of bacteremia or complications (Figure 5). Tables 1-2 provide the clinical and pathological criteria for the diagnosis of INAA, respectively.

**Table 1:** Clinical criteria for the diagnosis of INAA In this manuscript, the term IAA is used consistently in the text. INAA: Infectious native aortic aneurysm; IAA: infectious aortic aneurysm

Clinical Criteria	Description	Findings in the Present Case
Clinical presentation	Either pain, fever ≥38 ℃, sepsis and/or concomitant infection.	Met
Laboratory results	Elevated inflammatory markers (e.g., C-reactive protein, leukocytosis), and/or positive cultures.	Met
Imaging	Rapid expansion of aneurysm; saccular, multilobular, or eccentric aneurysms; peri-aortic gas; soft tissue mass or fluid; or multiple aneurysms with these features.	Met
Diagnosis based on criteria	Definite diagnosis: 3/3 clinical criteria met, with no alternative diagnosis more likely.	Definite diagnosis
	Probable diagnosis: 2/3 criteria met, with no alternative diagnosis more likely.	
	Not probable: only 1/3 criterion met.	

**Table 2:** Pathological criteria for the diagnosis of INAA In this manuscript, the term IAA is used consistently in the text. INAA: Infectious native aortic aneurysm; IAA: infectious aortic aneurysm

Pathological Criteria	Description	Findings in the Present Case
Surgical/Histological findings	Intraoperative finding of pus or abscess in the aneurysm wall, or positive microbiological culture/histology from guided aspiration, in a patient with clinical suspicion of INAA (definite or probable).	Met

## Discussion

Kp is a Gram-negative bacillus commonly implicated in urinary tract and respiratory infections [1,11]. hvKp has emerged as a significant pathogen, particularly in East Asia, and is associated with invasive syndromes, such as liver abscess, endophthalmitis, meningitis, and necrotizing fasciitis [1,11]. This increased virulence is attributed to the overproduction of capsular polysaccharides (notably K1 and K2 serotypes), enhanced siderophore secretion, and the presence of virulence genes such as magA and rmpA [2,12].

HvKp and cKp both produce penicillinase, making third-generation cephalosporins the usual first-line therapy [13]. However, the rising prevalence of ESBL-producing strains in Japan underscores the importance of performing susceptibility testing to guide appropriate antimicrobial selection [14].

While whole-genome sequencing (WGS) and PCR-based detection of virulence genes, such as rmpA and iucA, remain the gold standard [15], their limited availability in clinical settings due to cost and insurance constraints necessitates the use of alternative methods, and these were not performed in our case. The string test offers a practical and cost-effective diagnostic tool, defining a positive result as a mucoviscous string ≥5 mm when a bacterial colony is lifted. Despite its lower diagnostic accuracy compared to molecular methods, the test retains a relatively high sensitivity (89%) and specificity (91%) [3]. In our case, the isolate tested positive on the string test, indicating the hmKp, which is often used as a surrogate for hvKp in clinical contexts. HmKp strains exhibit enhanced tissue adherence and resistance to host immune clearance, contributing to their pathogenicity [1].

There are no established diagnostic criteria for IAA; however, the Delphi consensus document provides standardized criteria for case reporting, and our case fulfilled all of these items [16] (Tables 1-2).

Although *S. aureus *and *Salmonella *spp. are the most frequently implicated pathogens in IAAs, Kp has also been reported as a causative agent [5,6]. A previous small case series-based review reported that approximately 23% of Kp-associated IAAs were attributable to hvKp [4].

Unlike typical aortic aneurysms, which expand slowly at a rate of 2-3 mm/year [7], IAAs tend to progress rapidly, with a median enlargement of 11 mm over 69 days [7]. Previous reports of hmKp-related IAAs documented aneurysmal growth of 56 mm in eight days and 40 mm in 30 days, respectively [8,17]. In our case, the aneurysm expanded by 7 mm within a single week, which is consistent with these prior observations. Considering that hmKp bacteremia was confirmed and no other pathogens were identified, hmKp is the most plausible causative pathogen of the IAA.

The prognosis of IAA is significantly influenced by timely intervention. Antibiotic therapy alone is associated with an in-hospital mortality rate of approximately 55%, whereas combined medical and surgical approaches reduce mortality to 11% [18]. In our case, emergency EVAR was performed on day 24 to prevent aneurysmal rupture, followed by definitive OAR on day 31. This staged intervention strategy was essential in achieving clinical recovery [18].

Postoperatively, standard treatment involves 6-12 weeks of intravenous antimicrobial therapy, followed by oral agents as needed based on clinical status and inflammatory markers [18,19]. In our case, the patient’s recovery was uneventful, with no recurrent bacteremia or symptoms. However, mild CRP elevation persists, and the patient remains on oral levofloxacin therapy under close monitoring.

In addition to microbial factors, host-related risk factors such as vascular calcification may have facilitated the development of IAA in our patient. Vascular calcification plays a key role in the pathogenesis of aneurysms, including IAA [20,21]. While our patient had no diagnosed comorbidities such as diabetes, hypertension, or dyslipidemia, his significant smoking and alcohol use likely contributed to pronounced aortic calcification, providing a favorable environment for IAA development [22,23]. Taken together, these findings suggest that both microbial virulence (hmKp) and host-related factors (vascular calcification) may have synergistically contributed to the onset and rapid progression of IAA in this case.

## Conclusions

When Kp is isolated from blood cultures, performing a string test is essential to identify hmKp. A positive string test suggests the presence of hvKp, which is associated with metastatic infections, such as liver abscess, endophthalmitis, meningitis, necrotizing fasciitis, and, though rare, IAA. Clinicians should maintain a high index of suspicion for these complications, especially if clinical improvement is inadequate despite appropriate therapy. In such cases, repeat CECT is critical to detect occult or delayed complications, facilitating timely diagnosis and intervention.
